# Complex interactions among soil physicochemical interactions and microbial community associations with potato common scab severity

**DOI:** 10.3389/fpls.2026.1802004

**Published:** 2026-04-01

**Authors:** Bak Gyeryeong, Kim Jeom-Soon, Lee Jeong-Tae, Kim Yang-Min

**Affiliations:** Highland Agriculture Research Institute, National Institute of Crop Science, Rural Development Administration, Pyeongchang, Republic of Korea

**Keywords:** amplicon sequencing, physicochemical properties, potato common scab, random forest, soil microbiota

## Abstract

Potato common scab, a disease caused by the pathogenic *Streptomyces* species, produces cork lesions on tubers, leading to significant reductions in marketable yield and crop quality worldwide. Although the established correlation between soil physicochemical properties and microbial communities with disease severity, the interactive effects of these factors remain to be fully understood. In this study, we conducted a comprehensive, multi-year investigation of 124 potato fields in South Korea. This investigation was undertaken to elucidate the combined influence of soil properties and microbial communities on the development of common scab disease. An analysis of the soil physicochemical characteristics was conducted in conjunction with high-throughput 16s rRNA and ITS amplicon sequencing. The application of random forest modeling, based on soil physicochemical properties, exhibited a moderate predictive accuracy. Consistent with this, the importance feature of individual variables remained low, suggesting complex interactions among soil factors. The analysis of microbial communities revealed taxa that described a correlation with severity levels, thus distinguishing between distinct severity groups. Rhodanobacteraceae and Mortierellaceae were identified as indicators of low-severity fields, whereas Sphingomonadaceae, Chaetomiaceae, and Cystofilobasidiaceae were associated with high-severity fields. Despite the identification of several severity-associated soil and microbial features, no predominant factor explaining common scab severity was detected. This finding suggests that disease development is influenced by complex interactions among soil physicochemical conditions and microbial communities rather than by a single dominant driver.

## 1 Introduction

Potato (*Solanum tuberosum* L.) is a globally important crop. However, its production is influenced by a multitude of diseases that reduce tuber quality and marketability. Potato common scab, a disease caused by the pathogenic *Streptomyces* species, is one of the most economically significant tuber blemish diseases worldwide (Lankau et al., 2020; Gleń-Karolczyk et al., 2022). The disease manifests as superficial to deep pitted or raised lesions on tuber surfaces, which severely downgrade market value and limit processing suitability (Charkowski et al., 2020). In severe cases, common scab can reduce marketable yield from over 90% to less than 40%. Despite the several studies conducted on the subject, effective and consistent disease management remains elusive.

The physicochemical properties of soil have been identified as pivotal factors in the development of common scab. Specifically, empirical evidence has identified soil pH as the primary factor associated with increased disease severity. Elevated pH levels, defined as a pH greater than 5.5, have been observed to be consistently associated with increased disease severity across multiple regions (Lazarovits et al., 2007; Kim et al., 2012). A field trial conducted in Korea demonstrated that a decrease in soil pH to approximately 5.0 led to a substantial reduction in disease severity, from 61.1% to 22.8%. Furthermore, other soil properties, including organic matter, moisture regime, texture, and nutrient concentrations, have been implicated in disease development. However, the effects of these factors were inconsistent in different geographic regions (Lazarovits et al., 2007; Sagova-Mareckova et al., 2015; Marketa et al., 2021). These inconsistencies imply that soil physiochemical factors may interact in complex, context-dependent ways rather than exerting simple, additive effects. The development of common scab was influenced by environmental and climatic conditions. Notably, the temperature and soil moisture conditions during the tuber initiation and bulking stages have been observed to influence the expression of symptoms and the subsequent progression of the disease (Lankau et al., 2020). Climatic variability has the capacity to modify nutrient dynamics (Zhang and Wienhold, 2002). Consequently, the factor may partially elucidate the inconsistent relationships observed between individual soil properties and the common scab severity in different regions and years. Concurrently, advancements in high-throughput sequencing have elucidated the impact of soil and tuberosphere microbial communities on the severity of common scab. A multitude of comparative studies have identified distinct bacterial community structures in soils that are conductive to disease versus those that are disease-suppressive. Taxa such as Pseudomonadaceae, Bradyrhizobiaceae, and Paenibacillaceae have been enriched in soils that are suppressive (Kopecky et al., 2018; Kopecky et al., 2019). Fungal communities and micro-eukaryotes have also been shown to differ between suppressive and conductive soils, though shifts in bacterial communities are typically more strongly associated with disease suppression (Kopecky et al., 2019). The application of biological control amendments has been demonstrated to mitigate the severity of diseases by augmenting antagonistic microbial populations and modulating soil metabolite profiles (Tomihama et al., 2016; Cao et al., 2025). Nevertheless, the predictive power of microbial community composition for disease severity remains inconsistent across landscapes. Various studies have been indicated that the presence of pathogen in a given environment does not necessarily correlate with subsequent development of disease. This suggest the existence of other factors that may influence the activity of these pathogens, such as soil chemistry, microbial interactions, and host factors (Sagova-Mareckova et al., 2015; Sagova-Mareckova et al., 2016). In Korea, previous work documented the presence of multiple pathogenic *Streptomyces* species in Jeju and have found that bacterial community composition varies more by location and sampling time than by potato cultivar (Hong et al., 2003; Lee et al., 2021). However, integrated analyses linking soil properties, microbial communities, and disease severity across multiple regions remain limited. Notwithstanding these advances, there are still knowledge gaps. The majority of extant studies have predominantly concentrated on individual factors or modest collections of variables. Despite the efforts of researchers to elucidate the effects on soil pH and specific nutrients, the collective and synergistic effects of multiple soil properties remain to be fully elucidated (Lazarovits et al., 2007; Sagova-Mareckova et al., 2015; Lankau et al., 2020). Furthermore, although microbial community profiling has identified candidate taxa associated with suppression or conduciveness, the mechanistic links between community composition and disease outcomes remain unclear (Kopecky et al., 2018; Kopecky et al., 2019).

To address these knowledge gaps, a comprehensive multi-year field survey was conducted across four distinct regions of South Korea. The present study integrates soil physicochemical properties, as well as bacterial and fungal community profiling. The objectives of this study are to: 1) quantify the individual and collective contributions of soil properties to common scab severity using machine learning approaches, 2) characterize microbial community associations with disease. This study offers insights into the multifactorial nature of potato common scab in East Asia and the challenges of identifying transferable disease management strategies in complex soil-plant-microbe systems.

## 2 Materials and methods

### 2.1 Study sites and experimental design

A comprehensive field survey was conducted over three consecutive growing seasons (2020, 2021, and 2022). A total of 124 potato production fields in four cultivation regions in South Korea: Gangneung, Jeju, Milyang, and Pyeongchang. These regions are distinguished by a broad spectrum of climatic conditions. Fields were managed in accordance with standard regional cultivation practices. The cultivated potato cultivars varied among different fields, in accordance with local grower preferences and market demands. At the time of harvest, or immediately thereafter, surveys were conducted to assess the severity of potato common scab in each field. The assessment of the disease was conducted through a visual examination of samples of tuber from each field. Tubers were sampled by harvesting five potato plants from each of three ridges within the field. The severity of common scab was determined for individual tuber using a five-point scale, with the percentage of tuber surface area affected by scab lesions: 0 = no visible lesions; 1 = minor lesions covering < 5% of tuber surface; 2 = lesions covering 5-10% of tuber surface; 3 = lesions covering 10-20% of tuber surface; 4 = severe lesions covering > 20% of tuber surface. According to the disease severity assessment, soil samples were collected from areas corresponding to two distinct severity classes: high-severity zones (disease severity score 4, >20% tuber surface affected) or low-severity zones (disease severity scores below 2, <10% tuber surface affected). Lesion morphology was not recorded in this survey and therefore was not considered in the severity classification. An effort was made to balance the sampling across severity classes. Our sampling strategy prioritized independent fields representing contrasting severity levels within each region. When this was not feasible due to field availability or uniform disease occurrence, sampling locations representing different severity levels were selected within the same field but separated by at least 7 m to minimize spatial overlap between sampling points. A total of 65 high-severity samples and 54 low-severity samples were collected from the 119 fields. The number of surveyed fields classified as low or high severity varied among regions due to differences in disease occurrence and field availability during the survey period. The regional distribution of surveyed fields according to severity class in shown in **Supplementary Figure 1**. The objective of this sampling approach was designed to capture the soil property and microbial community gradients associated with contrasting disease outcomes across diverse production environments. Soil samples were obtained from the rhizosphere-influenced bulk soil. The specific sampling protocols varied by analytical target and are detailed in the respective subsections below.

### 2.2 Soil texture and physical properties

In order to facilitate the analysis of soil texture, a composite soil sample was collected from each field. The measurement of soil particle size distribution was conducted using the hydrometer method following dispersion with sodium hexametaphosphate. The proportions of sand (2.0-0.05 mm), silt (0.05-0.002 mm), and clay (<0.002 mm) were measured, and soil texture classes were assigned according to the USDA soil texture triangle classification system. An analysis of the soil physical properties was conducted using samples collected from two depth intervals: topsoil (5–10 cm) and subsoil (20–25 cm). To ensure the accuracy of the measurements, three replicate undisturbed soil cores were collected from each depth interval per field. These cores were obtained using a stainless steel core sampler with an internal volume of 100 cm^3^. The cores were carefully inserted vertically into the soil profile to minimize structural disturbance, and both ends were promptly sealed to preserve field moisture conditions during transport. In the laboratory, soil bulk density was determined gravimetrically by measuring the fresh weight of each core sample, followed by oven-drying at 105 °C for 48 h to constant weight. Bulk density was calculated as the ration of oven-dried soil mass (g) to core volume (cm^3^). Total soil porosity was subsequently calculated from bulk density values using an assumed particle density of 2.65 g/cm3, according to the following equation:

Porosity (%) = [1 − (bulk density/particle density)] × 100.

### 2.3 Soil chemical properties analysis

Although 124 fields were surveyed, soil physicochemical data were available for 113 fields due to sample availability. Soil samples were collected from a depth of 5–15 cm using a soil knife. Six subsamples were collected from each field, placed into a single plastic bag, and thoroughly mixed to create a composite sample representative of the field. Soil sampling was conducted within the same zones used for disease severity assessment. The composite soil sample was homogenized and divided into two subsamples for chemical analysis and DNA extraction. One subsample was transferred to the laboratory, air-dried at room temperature for 48 h, gently crushed, and passed through a 2 mm sieve prior to analysis. Soil pH was measured electrometrically in a 1:5 (w/v) soil:deionized water suspension after 30 min of equilibration with intermittent shaking. pH was determined using a calibrated pH meter (Thermo Scientific, Waltham, MA, USA). Soil organic carbon (OC) was determined by dry combustion using a C/N elemental analyzer (Vario Max Cube, Elementar Analysesysteme GmbH, Langenselbold, Germany), and organic matter (OM) content was calculated from OC values using the van Bemmelen conversion factor (OM = OC x 1.724). Available phosphorus was extracted using the Lancaster method and subsequently quantified colorimetrically via an UV/VIS spectrophotometer (Lambda 25, PerkinElmer Inc., Norwalk, CT, USA) at 880 nm. Exchangeable cations (K, Ca, Mg) and additional elements (S, Al, Mn) were extracted with 1 M ammonium acetate (NH4OAC) solution at pH 7.0 (1:10 soil:extractant ratio) and determined by inductively coupled plasma optical emission spectrometry (ICP-OES; Optima 2100DV, PerkinElmer Inc., Norwalk, CT, USA). All chemical analyses were performed in triplicate for each soil sample.

### 2.4 Statistical analyses using soil physicochemical properties and disease severity

Soil texture were visualized using USDA soil texture triangle to classify samples according to their sand, silt, and clay proportions. The texture triangle plot was generated using the **soiltexture** package in R (version 4.3.3). Non-metric multidimensional scaling (NMDS) was performed to visualize to multivariate relationships among soil physical and chemical properties and to assess their associations with common scab severity. The analysis included soil porosity characteristics, bulk density, soil texture components, and chemical properties. NMDS ordination was conducted using the **vegan** package in R. Dissimilarity among samples was calculated using the Bray-Curtis distance metric.

A random forest classification model was developed to predict potato common scab severity classes based on soil physical and chemical properties. The model was implemented in Python (version 3.9) using the **scikit-learn** library with default parameters. Input features included soil texture components, nutrient contents, soil pH, and porosity-related variables. Given that three replicate samples were subjected to chemical analysis for each field, model performance was evaluated using field-blocked across-validation, in which all replicate samples from a given field were assigned to the same validation fold. This approach ensured that replicate samples from the same field did not appear simultaneously in the training of the testing datasets. The model performance was evaluated using several metrics, including classification accuracy, balanced accuracy, sensitivity, specificity, and the area under the receiver operating characteristic curve (ROC-AUC). Feature importance was quantified using mean decrease in impurity (Gini importance).

To explore the interrelationships among soil variables associated with common scab severity, a correlation network analysis was performed using the top 10 most important feature identified by the random forest model. Pairwise Pearson correlation coefficients were calculated among these selected soil properties. To focus on meaningful associations and reduce network complexity, only correlations with an absolute value greater than 0.3 were retained for network construction. The correlation network was constructed and visualized using the NetworkX library (version 3.1) in Python. In the network graph, nodes represent individual soil variables, and edges represent correlations between variables. Edge color indicates the direction of correlation: blue edges denote positive correlations, and red edges denote negative correlations. Node size was scaled according to degree centrality, defined as the number of edges connected to each node, reflecting the relative connectivity and potential importance of each variable within the network structure.

### 2.5 DNA extraction and amplicon sequencing

For microbial community analysis, bulk soil samples were collected from each field and immediately placed in plastic bags on ice for transport to the laboratory. In the laboratory, soil samples were passed through an ethanol-sterilized 2 mm sieve and thoroughly homogenized to ensure sample uniformity. Five replicate subsamples were then taken from each homogenized soil sample for DNA extraction. Total genomic DNA was extracted using the ISOIL II DNA extraction kit (Nippon Gene Co., Ltd., Tokyo, Japan) following the manufacturer’s protocol. Extracted DNA quality and concentration were assessed using a NanoDrop spectrophotometer (Thermo Fisher Scientific, Waltham, MA, USA), and DNA samples were stored at -20 °C prior to PCR amplication.

Bacterial and fungal communities were characterized by amplicon sequencing of the 16s rRNA gene and internal transcribed spacer (ITS) region, respectively. For bacterial community profiling, the V4 hypervariable region of the 16S rRNA gene was amplified using the primer pair 515F (5’-TCGTCGGCAGCGTCAGATGTGTATAAGAGACAG-GTGCCAGCMGCCGCGGTAA-3’ (Turner et al., 1999)) and 806R (5’-GTCTCGTGGGCTCGGAGATGTGTATAAGAGACAG-GGACTACHVGGGTWTCTAAT-3’ (Apprill et al., 2015)) following the Illumina 16S metagenomic sequencing library preparation protocol (Illumina, Inc., San Diego, CA, USA). These primers include Illumina adapter overhang sequences for downstream library preparation. For fungal community profiling, the ITS1 region (Gardes and Bruns, 1993) was amplified using primers ITS1F (5’-TCGTCGGCAGCGTCAGATGTGTATAAGAGACAG-CTTGGTCATTTAGAGGAAGTAA-3’) and ITS2R (5’-GTCTCGTGGGCTCGGAGATGTGTATA AGAGACAG-GCTGCGTTCTTCATCGATGC-3’), which also include Illumina adapter sequences.

PCR amplification was performed using AmpliTaq Gold DNA Polymerase (Applied Biosystems, Foster City, CA, USA). The thermal cycling conditions were as follows: initial denaturation at 95 °C for 10 min; 30 cycles of denaturation at 95 °C for 30 s, annealing at 55 °C for 30 s, and extension at 72 °C for 30 s; followed by a final extension at 72 °C for 7 min. PCR products were verified by agarose gel electrophoresis and purified using AMPure XP beads (Beckman Coulter, Brea, CA, USA). Purified amplicons were indexed using the Nextera XT Index Kit (Illumina, Inc., San Diego, CA, USA) to enable sample multiplexing. Indexed libraries were quantified using an Agilent 2100 Bioanalyzer (Agilent Technologies, Santa Clara, CA, USA). Libraries were pooled in equimolar concentrations and sequenced on an Illumina Miseq platform (Illumina, Inc., San Diego, CA, USA) using 2 x 300 bp paired-end sequencing chemistry at Macrogen Inc. (Seoul, South Korea). Raw sequence data were demultiplexed and provided as paired-end FASTQ files.

### 2.6 Bioinformatics and sequence data processing

Raw paired-end sequence reads were processed using CLC Workbench (version 24.0, QIAGEN, Aarhus, Denmark). Quality filtering and denoising procedures were applied to identify amplicon sequence variants (ASVs). Taxonomic assignment of bacterial 16S rRNA gene sequences was performed using the SILVA SSU reference database (version 138.1), while fungal ITS sequences were classified using the UNITE database (version 9.0). To reduce noise from rare or potentially spurious sequences, stringent abundance filtering was applied. The initial bacterial dataset comprised 441,311 ASVs, while the fungal dataset contained 19,959 ASVs. To eliminate low-abundance features that likely represent sequencing noise, ASVs were filtered based on the minimum total read counts across all samples. For bacteria, ASVs with fewer than 100 total reads were removed, resulting in 38,772 ASVs. For fungi, ASVs with fewer than 50 total reads were removed, resulting in 2,743 ASVs. Because the bacterial dataset still contained a large number of ASVs, downstream multivariate analyses were conducted using the 5,000 most prevalent bacterial ASVs across all samples. This step reduced dataset sparsity and improved computational efficiency while retaining the dominant community signal (**Supplementary Table 1**). Because sequencing depth varied among samples, ASV counts were converted to relative abundances prior to downstream analyses.

Bacterial and fungal community alpha diversity was assessed using phyloseq and microbiome packages in R (version 4.3.3.). Three complementary metrics were calculated for each sample: observed ASV richness, Shannon diversity index, and inverse Simpson index. Differences in alpha diversity between high-severity and low-severity groups were visualized using boxplots generated with the ggpubr package and statistically evaluated using Welch’s t-test. Statistical significance was set at α = 0.05.

Beta diversity was assessed to evaluate compositional dissimilarity among microbial communities using weighted UniFrac distance matrices. Principal coordinates analysis (PCoA) was performed to visualize community dissimilarity patterns according to geographic region and common scab severity using the phyloseq package in R. Permutational multivariate analysis of variance (PERMANOVA) was conducted to test the effect of potato common scab severity on bacterial and fungal community composition using the vegan package in R. Community dissimilarity was calculated using Bray-Curtis distance, and statistical significance was assessed using 999 permutations.

An indicator ASV analysis was performed using labdsv package in R to identify bacterial and fungal taxa significantly associated with high- or low- severity fields. The detected indicator ASVs were visualized as cumulative relative abundance, depending on common scab severity, using ggplot2 in R.

Canonical correspondence analysis (CCA) was conducted to examine the relationships between microbial community composition, soil physicochemical properties, and common scab severity. Prior to ordination, ASV abundance tables were normalized to relative abundance to account for differences in sequencing depth among samples. CCA was conducted using the **vegan** package in R, with microbial community composition as the response matrix and soil physicochemical variable as explanatory variables. Explanatory variables included soil texture fractions, pH, bulk density, organic matter content, porosities, and nutrient concentrations. Potato common scab severity (high and low) was not included as a constraining variable but was overlaid onto the ordination as a categorical grouping factor. Ordination biplots were generated using the ggplot2 package.

## 3 Results

### 3.1 Soil physicochemical properties and their relationships with common scab severity

A study was conducted in which soil samples were collected from 124 potato fields across four distinct climate regions in South Korea over a period of three years. The results of the study indicated that the soil samples exhibited diverse physicochemical characteristics. Soil texture analysis revealed substantial variability across the various sampling sites, with the samples distributed across multiple USDA soil texture classes (**Supplementary Figure 2**). Soils from Gangneung were predominantly sandy loam, while those from Pyeongchang ranged from sandy loam to loam. In contrast, soils from Jeju and Milyang were predominantly classified as silt loam. The analysis revealed no discernible patterns in soil texture that were associated with the disease incidence, suggesting that soil texture alone does not function as a primary determinant of disease incidence.

**Figure 1:**
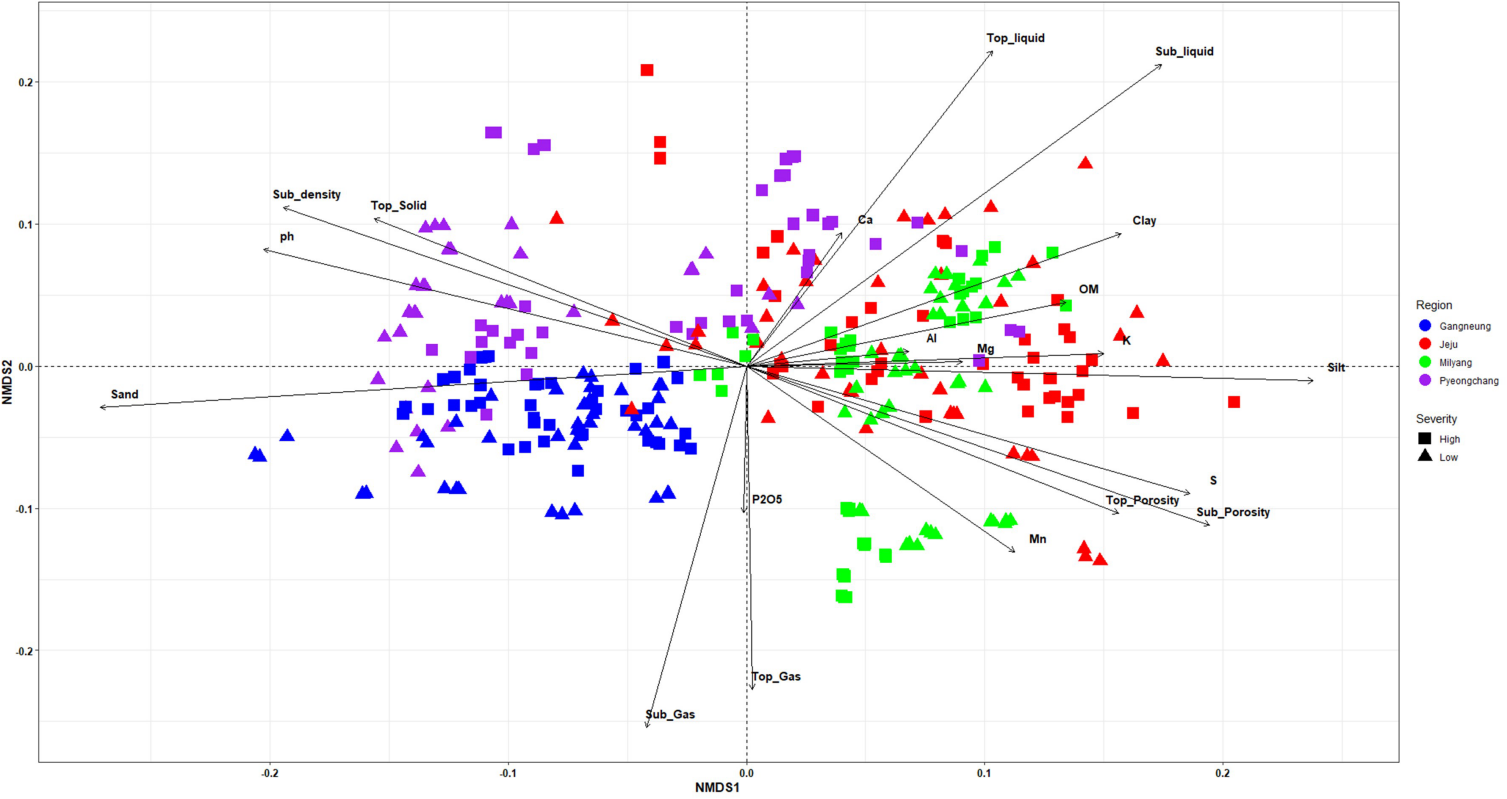
Non-metric multidimensional scaling (NMDS) ordination of soil samples based on soil physical and chemical properties. Arrows represent soil, indicating the direction and strength of correlations with the ordination axes. Colors indicate sampling regions, and symbols represent severity levels.

Non-metric multidimensional scaling (NMDS) ordination based on soil physical and chemical properties revealed considerable heterogeneity among samples (**Figure 1**). Compared to other regions, samples from Gangneung exhibited a relatively clustered distribution, in contrast to the dispersed distribution observed in Jeju samples. Furthermore, no discernible clustering was identified between the high and low severity groups. Preliminary findings suggest that environmental vectors are associated with variations in soil properties. These variations are influenced by multiple soil variables, including nutrient contents, texture components, and porosity-related parameters. However, the lack of clear separation between severity groups is a limitation.

**Figure 2:**
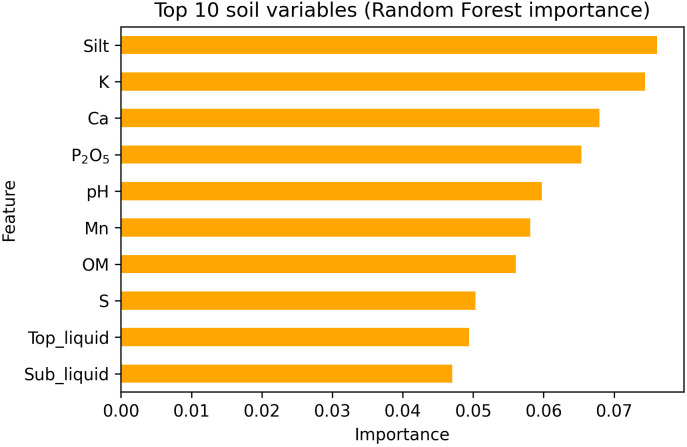
Variable importance of the top 10 soil physicochemical properties identified by the Random Forest model used to explore associations with potato common scab severity.

In order to identify the most influential soil variables associated with common scab severity, a random forest classification analysis was conducted using 22 soil physical and chemical variables as predictors. When evaluated using field-blocked cross-validation to account for replicate samples collected from the same field, the RF model demonstrated moderate classification performance (accuracy: 0.63, balanced accuracy: 0.68, ROC-AUC: 0.75). The findings suggest that soil physicochemical variables exhibit limited capacity for distinguishing between fields with high and low common scab disease severity. Preliminary feature importance analysis identified several soil variables that contributed relatively more to classification performance. Among the measured variables, silt content, exchangeable potassium (K), calcium (Ca), available phosphorus (P_2_O_5_), and soil pH showed comparatively higher importance scores in the RF model. However, the importance values of individual predictors were relatively small, with the highest score being 0.076 (**Figure 2**), indicating that no single soil property strongly determined common scab severity.

Notably, the top 10 influential factors associated with common scab disease were not consistent across regions and showed region-specific patterns (**Supplementary Figure 3**). Among these variables, manganese (Mn) consistently exhibited higher concentrations in samples with lower severity across all four regions. However, given the fact that Mn is a trace element, further discussion and validation are required.

**Figure 3:**
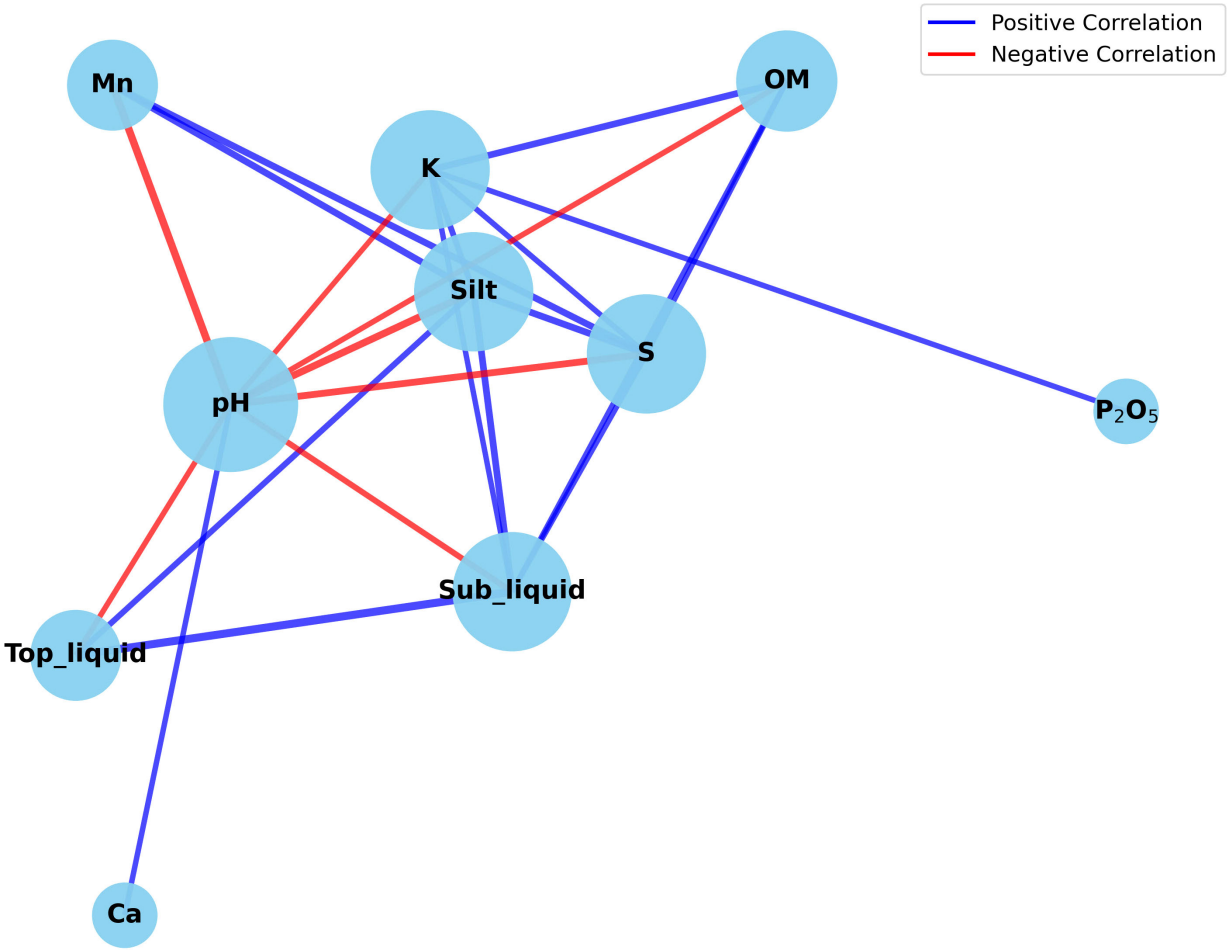
Correlation network of key soil variables associated with potato common scab severity. Node represent soil properties selected from the random forest analysis, with node size scaled by degree centrality. Edges describe Pearson correlations with an absolute value greater than 0.3. Blue and red edges represent positive and negative correlations, respectively.

Correlation network analysis was performed on a set of key soil variables selected from the random forest model. This analysis revealed intricate relationships among soil properties (**Figure 3**). The network displayed multiple significant correlations (|r| > 0.3), with both positive and negative associations among texture components, nutrient contents, and other physicochemical parameters. Several variables related to soil texture and moisture status, including silt content, soil sulfur (S), exchangeable potassium (K), and subsoil liquid-filled porosity, formed a relatively well-connected cluster within the network. Furthermore, soil pH exhibited multifaceted associations with other soil variables. Conversely, the available phosphorus (P_2_O_5_) showed a comparatively lower number of connections with other variables. These patterns suggest that multiple soil physicochemical properties are interconnected, rather than operating as independent factors. Overall, the correlation network provides a descriptive overview of relationships among soil variables associated with common scab severity.

### 3.2 Microbial community composition and diversity in relation to common scab severity

Following quality filtering, samples with low sequencing depth were excluded from further analyses of the microbial community. The final numbers of samples retained for bacterial and fungal datasets across regions and severity levels are summarized in **Supplementary Figure 4**. The microbial community structure was compared between common scab severity classes using PERMANOVA analysis. The result revealed statistically significant differences in bacterial and fungal community composition between potato common scab severity levels (**Table 1**). However, the proportion of variance explained by disease severity was very samll, accounting for only 0.2% and 0.7% of the total variation in bacterial and fungal communities, respectively. These results indicate that while severity-related differences were statistically detectable, disease severity represents only a minor component of the overall microbial community variation observed across fields.

**Table 1:** PERMANOVA results for bacterial and fungal communities by potato common scab severity.

Kingdom		Df	Sum of squares	R^2^	F	Pr(>F)
Bacteria	Severity	1	0.639	0.00228	1.3106	***
	Residual	574	279.905	0.99772		
	Total	575	280.544	1.00000		
Fungi	Severity	1	1.489	0.00784	3.752	***
	Residual	475	188.489	0.99216		
	Total	476	189.978	1.00000		

The bacterial diversity indices did not differ significantly between the high and low severity groups (**Supplementary Figure 5A**). In fungal communities (**Supplementary Figure 5B**), a statistically significant divergence was observed on the observed ASVs and the inverse Simpson index between the severity groups. However, the Shannon index showed an absence of statistically significant variation. Despite these statistical disparities, the overall magnitude of variation in fungal diversity between the severity groups was limited. When examined separately across regions (**Supplementary Figure 6**), the relationship between disease severity and microbial diversity was not consistent. These results suggest that no discernible relationship between disease severity and microbial diversity was observed across regions.

Principal coordinates analysis (PCoA) based on weighted UniFrac distances revealed regional clustering patterns for both bacterial (**Supplementary Figure 7**) and fungal (**Supplementary Figure 8**) communities, but no clear separation between severity groups. This pattern aligns with the NMDS results for soil properties, reinforcing the conclusion that common scab severity is not associated with distinct, severity-specific microbial community profiles at the whole-community level.

### 3.3 Indicator taxa associated with common scab severity

**Figure 4:**
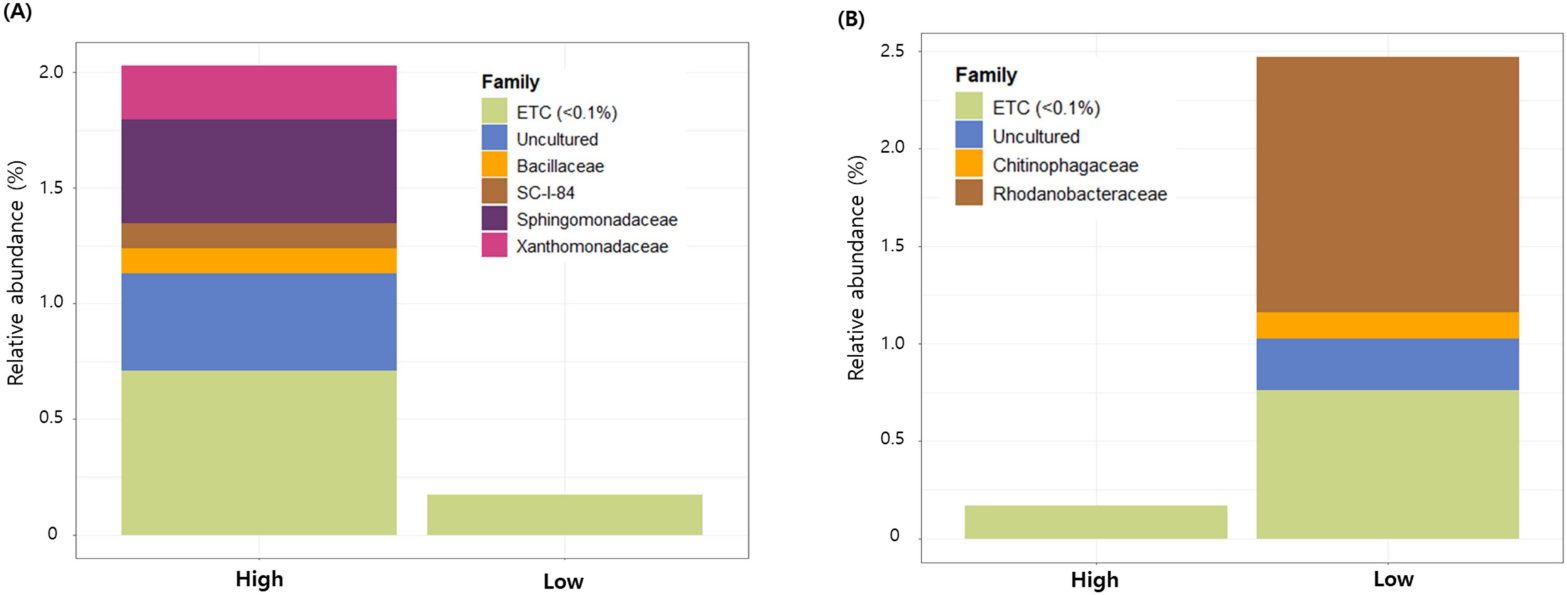
Relative abundance of bacterial indicator ASVs associated with high **(A)** and low **(B)** potato common scab severity.

Despite the absence of clear whole-community differentiation, indicator species analysis identified several bacterial and fungal ASVs that were statistically associated with high and low severity conditions. A total of 104 and 140 bacterial indicator ASVs were identified with the high- and low-severity groups, respectively (**Figure 4**). In the high-severity group, indicator ASVs were predominantly affiliated with the families Sphingomonadaceae and Xanthomonadaceae, whereas indicator ASVs associated with the low-severity group were mainly classified within the family Rhodanobacteraceae. However, the indicator ASVs collectively accounted for a small proportion of the total community, comprising approximately 2.0–2.5% of the total relative abundance.

**Figure 5:**
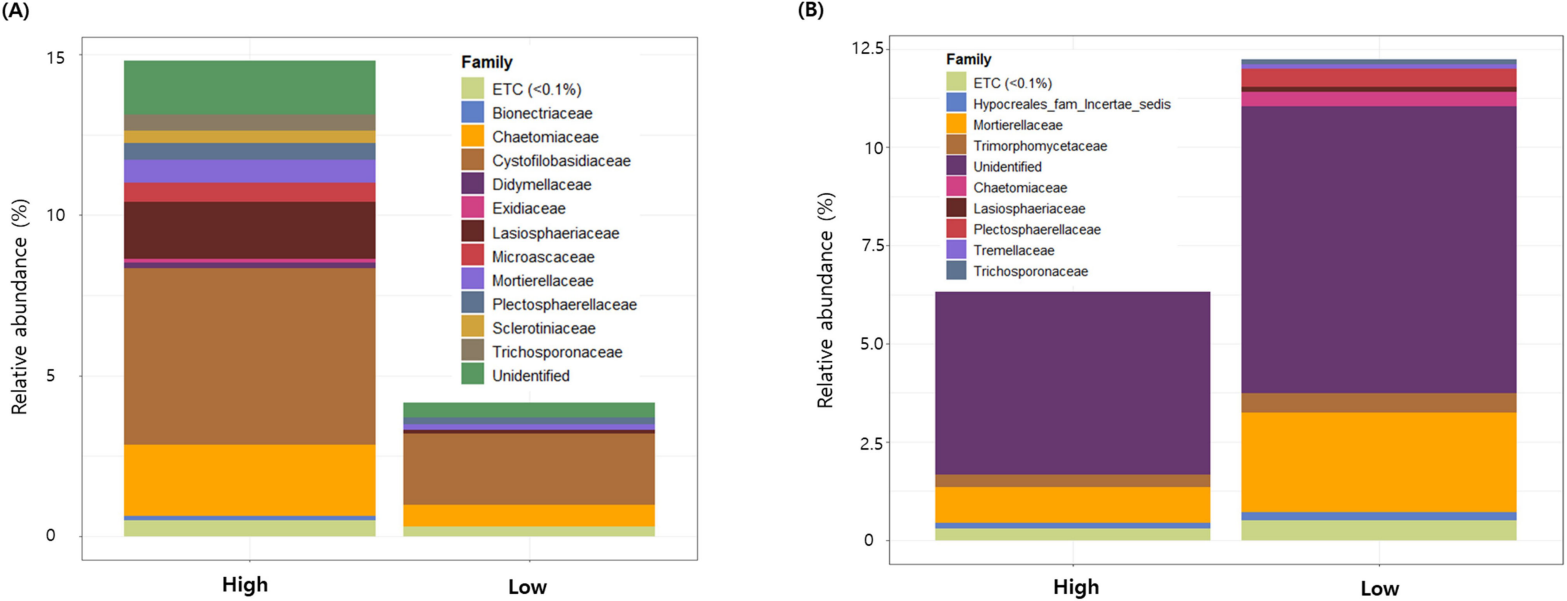
Relative abundance of fungal indicator ASVs associated with high **(A)** and low **(B)** potato common scab severity.

For fungal communities, a total of 316 and 246 indicator ASVs were identified with the high- and low-severity groups, respectively, representing approximately 12.5-15% of the total relative abundance (**Figure 5**). Indicator ASVs associated with the high-severity group were predominantly affiliated with the families Chaetomiaceae, Cystofilobasidiaceae, and Lasiosphaeriaceae. Conversely, indicator ASVs linked to the low-severity group were predominantly represented by members of the family Mortierellaceae.

Given the well-established role of *Streptomyces* species as the primary causal agents of potato common scab, we further examined the relative abundance of the *Streptomyces* genus across severity groups. While *Streptomyces* was detected in samples from all regions and severity levels, no significant difference in overall *Streptomyces* abundance was observed between high and low severity groups (**Supplementary Figure 9**). Moreover, no *Streptomyces* ASVs were identified as indicator taxa for the high-severity group. Because genus-level classification does not distinguish pathogenic from non-pathogenic Streptomyces strains, these results should be interpreted cautiously. Together, these findings suggest that the presence or relative abundance of the Streptomyces genus alone may not adequately explain variation in disease severity.

### 3.4 Relationships between microbial communities and soil physicochemical properties

Canonical correspondence analysis (CCA) was performed to assess the relationships between microbial community composition and soil physicochemical variables (**Supplementary Figures 10**, **11**). The analysis revealed that neither the bacterial nor the fungal communities exhibited a clear separation between the disease severity groups. It has been demonstrated that, in a consistent manner, PERMANOVA and β-diversity analyses have indicated that disease severity has only explained only a minor proportion of the variation in microbial communities. Furthermore, random forest analyses based on soil physicochemical variables also demonstrated low predictor importance. The CCA revealed associations between microbial communities and soil properties, but no distinct clustering by disease severity was observed for either bacterial or fungal communities. These results are consistent with the preceding analyses and further confirm that disease severity is not explained by single soil or microbial factors alone.

## 4 Discussion

The soil physicochemical properties exhibited substantial heterogeneity across diverse potato cultivation regions. Furthermore, no singular soil factor or texture class was identified as a dominant factor influencing the severity of common scab. Although the established role of soil pH and moisture-related factors in development of common scab (Lazarovits et al., 2007; Gudmestad and Johnson, 2008), our findings suggest that these factor alone are inadequate to account for the observed variation in disease incidence across different regions. The absence of severity-associated clustering in NMDS ordinations and the balanced feature importance observed in random forest models suggest that disease outcomes are not driven by isolated soil properties, but rather by complex combinations of multiple interacting factors. The moderate predictive performance of the random forest model further supports this interpretation. Inconsistencies reported in previous studies regarding the influence of specific soil parameters across regions and environmental conditions may reflect this underlying complexity (Davis et al., 1976; Lambert et al., 2005; Lazarovits et al., 2007; Dees and Wanner, 2012; Sagova-Mareckova et al., 2015; Kopecky et al., 2021). The correlation network analysis of key soil variables reinforces this interpretation by revealing strong interconnections among soil texture, moisture-related parameters, organic matter, and nutrient availability. Silt content, liquid-filled porosity, sulfur, and potassium were found to be interconnected hubs, indicating that integrated soil physical and chemical conditions may collectively influence disease development. This pattern aligns with the findings of previous studies, which demonstrated that soil texture influences disease severity in conjunction with related soil chemical conditions, particularly those involving organic matter, potassium, and pH (Dees and Wanner, 2012; Lankau et al., 2020; Kopecky et al., 2021). Soils with higher silt content are tend to exhibit greater stability in terms of nutrient and moisture availability (Ye et al., 2024; Mishra and Singh, 2025). These differences in soil physical stability, driven by its texture, may indirectly influence the development of common scab by establishing environments that favor or suppress disease occurrence. This influence is more likely to occur through the establishment of these environments rather than through the texture’s direct effects alone.

Manganese concentrations tended to be higher in low-severity fields across regions. Previous studies have reported potential associations between Mn availability and reduced common scab severity (McGregor and Wilson, 1966; Lazarovits et al., 2007). However, responses to Mn availability have been shown to vary depending on soil conditions, microbial activity, and management practices (Barnes, 1972; Kopecky et al., 2021). Because, Mn is a trace element whose bioavailability is strongly influenced by soil chemical and redox conditions, the observed association should be interpreted cautiously. The present study does not provide experimental evidence supporting a direct suppressive role of Mn in common scab development. Therefore, further experimental validation is necessary to determine its functional role in common scab suppression. The findings suggest that soil physicochemical properties contribute to common scab severity primarily through their interactive and cumulative effects, rather than through single dominant soil constraints.

Despite the statistically significant differences in bacterial and fungal community composition between common scab severity groups, disease severity explained only a small proportion of the overall variation in microbial communities. This limited explanatory power, in conjunction with the absence of severity-associated clustering in PCoA and CCA ordinations, indicates that common scab severity is not driven by large-scale shifts in microbial community structure. Instead, microbial assemblages appear to be shaped primarily by regional factors and environmental heterogeneity, with disease severity exerting a comparatively weak influence at the whole-community level. A similar pattern has been observed in potato common scab studies. In these studies, the composition and function of the soil microbiome in the geocaulosphere soil have been associated with disease severity. However, these studies have not yielded a strong separation of the overall community structure between severity groups (Shi et al., 2019; Shi et al., 2025). Despite the modest magnitude of whole-community differentiation was small, indicator taxa identified several bacterial and fungal taxa associated with disease severity. However, these taxa collectively accounted for only a small fraction of the total community, particularly for bacteria, suggesting that severity-associated microorganisms are numerically rare taxa rather than dominant community members.

The absence of *Streptomyces* ASVs, as indicated by paucity of its occurrence, lends further support to this interpretation (Ryan and Kinkel, 1997; Shi et al., 2025). Given that pathogenic and non-pathogenic strains cannot be distinguished at the genus level using amplicon sequencing, total *Streptomyces* abundance alone is unlikely to reliably reflect pathogen pressure. These findings highlight the limitations of genus-level taxonomic resolution for interpreting pathogen-microbiome interactions in common scab development.

The relationships between microbial communities and soil physiochemical properties further enhance this complexity of dynamic system. Although the fact that CCA revealed associations between microbial composition and multiple soil variables, neither bacterial nor fungal communities showed clear patterns in constrained ordination space that could be attributed to severity. This finding is consistent with previous studies showing that common scab severity is not determined by overall microbial community structure or pathogen abundance along (Lankau et al., 2020; Kopecky et al., 2021; Shi et al., 2025). Earlier work demonstrated a correlation between compositional changes in microbial communities surrounding potato tubers and disease outcomes. This correlation persists even when community-wide diversity metrics do not exhibit significant differences (Shi et al., 2019). Subsequent studies further emphasized that soil management practices and physicochemical conditions indirectly influence common scab severity, rather than through direct effects on specific taxa (Kopecky et al., 2021). Thus, the findings of these studies support to the interpretation that soil conditions broadly shape the assembly of microbial communities. By contrast, the severity of a disease is determined by context-dependent interactions among soil properties, microbial functional potential, and host response processes. These interactions are not readily captured by community-level ordination alone.

Another limitation of this study is that variation in potato cultivars and lesion morphology was not incorporated into the analyses. Potato cultivars are known to differ in their susceptibility to common scab, and interactions between host genotype and pathogenic Streptomyces species can influence disease expression and microbial communities. In addition, different lesion types may reflect distinct pathogenic strains or infection processes. Because the present field survey focused on severity indices based on lesion coverage rather than lesion morphology, these factors could not be evaluated in the current analyses (Ismail et al., 2020; Ghuffar et al., 2025). Further studies integrating cultivar identity, lesion type, and pathogen strain diversity may help further clarify the mechanisms underlying variability in common scab severity across ecologically different environment.

These findings are also consistent with emerging perspectives in soil health research, which emphasize that soil-borne disease outcomes often arise from interactions among soil physicochemical conditions, microbial community structure, and management practices rather than from single dominant drivers. Recent conceptual advances highlight the importance of integrating soil physicochemical properties with microbiome functionality to support sustainable soil management and disease suppression in agricultural systems (Risoli et al., 2026).

Overall, this study highlights the multifactorial nature of potato common scab in field environments. Neither soil physicochemical variables nor microbial community structure alone illustrated strong explanatory power for disease severity across the surveyed fields. Instead, the results suggest that common scab severity likely reflects complex interactions among soil physical conditions, nutrient availability, and microbial community composition. Collectively, these results underscore the complexity of the soil-microbe-plant interactions that underlie potato common scab and highlight the importance of integrative approaches.

## Data Availability

The sequencing data generated in this study have been deposited in the European Nucleotide Archive (ENA) under accession numbers ERR16151829-ERR16153006, comprising 578 bacterial (16S rRNA gene) and 600 fungal (ITS) amplicon sequencing datasets. Samples that failed PCR amplification or did not meet quality control criteria during sequence processing were excluded.
