# Supplementary material for: Complex interactions among soil physicochemical interactions and microbial community associations with potato common scab severity
Source: Front Plant Sci. 2026 Apr 1;17:1802004. doi: 10.3389/fpls.2026.1802004 (PMC13079313; doi:10.3389/fpls.2026.1802004)

Supplementary Material

# Supplementary Data

Supplementary Material should be uploaded separately on submission. Please include any supplementary data, figures and/or tables.

Supplementary material is not typeset so please ensure that all information is clearly presented, the appropriate caption is included in the file and not in the manuscript, and that the style conforms to the rest of the article.

# Supplementary Figures and Tables

For more information on Supplementary Material and for details on the different file types accepted, please see [here](https://www.frontiersin.org/guidelines/author-guidelines#supplementary-material).

## Supplementary Figures


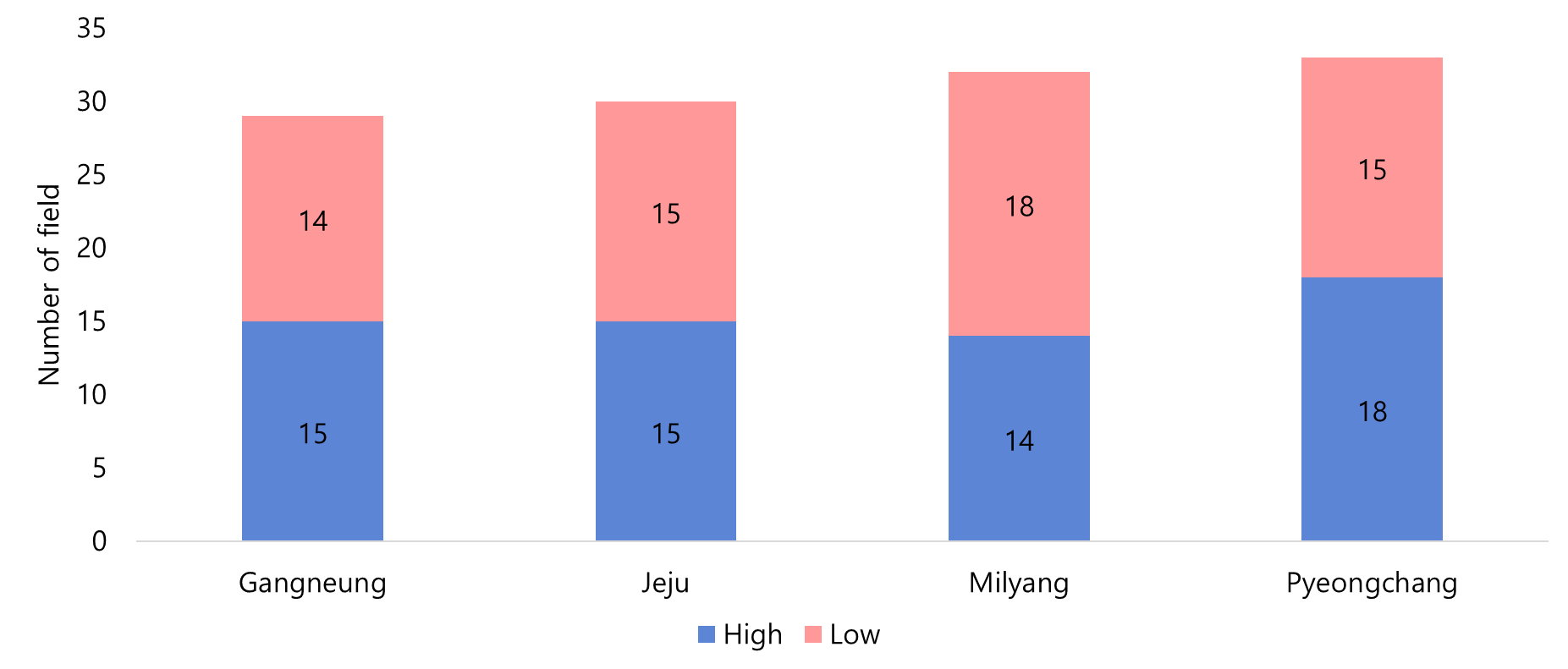


Supplementary Figure S1. Regional distribution of potato fields according to common scab severity.

Supplementary Figure S2. Soil texture classification of samples based on the USDA soil texture triangle. The percentages of sand, silt, and clay were used to classify soil texture classes. Each point represents an individual soil sample, with colors indicating sampling regions and point sizes representing severity levels (high and low). Shaded areas denote USDA soil texture classes.


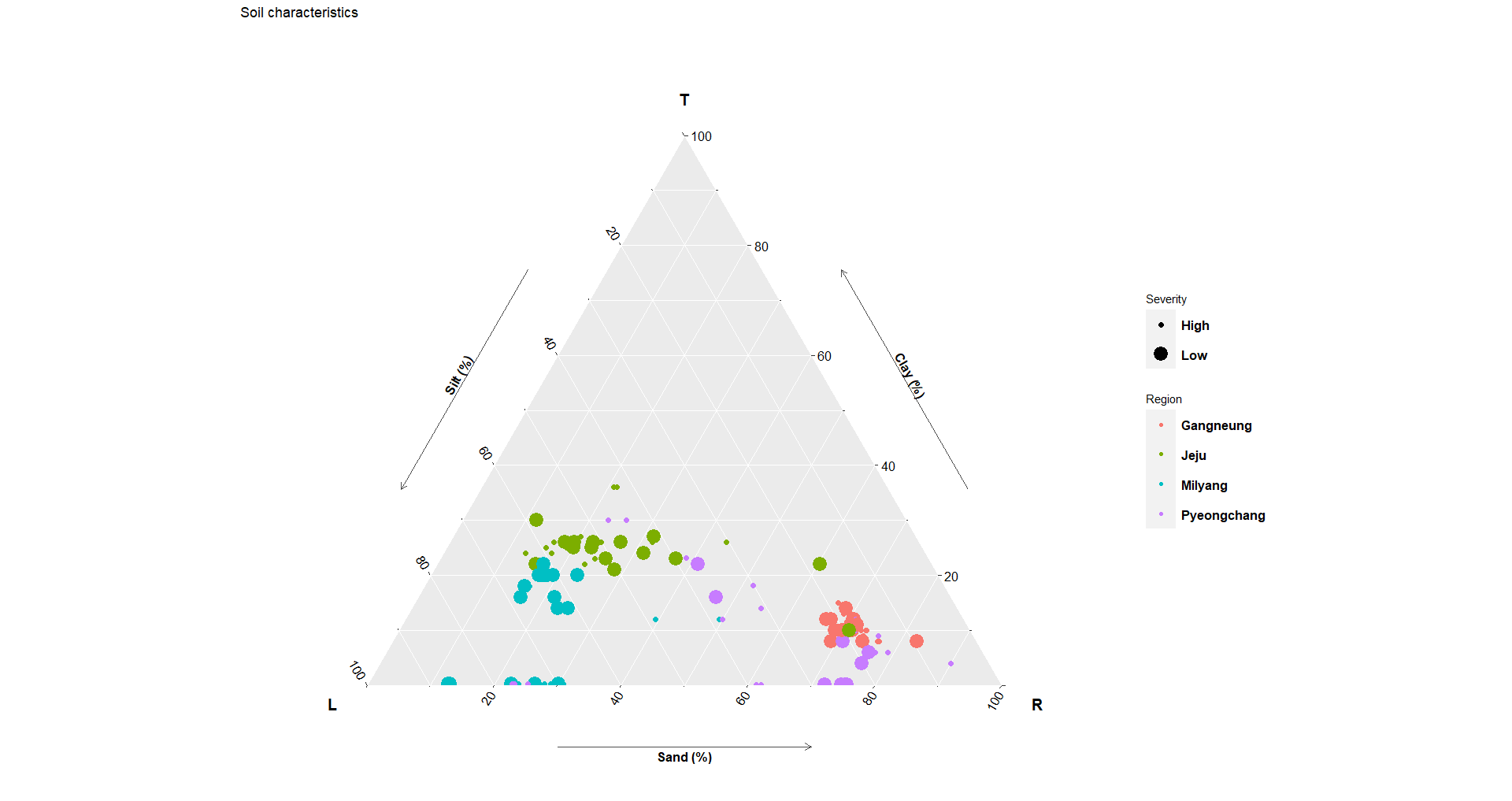

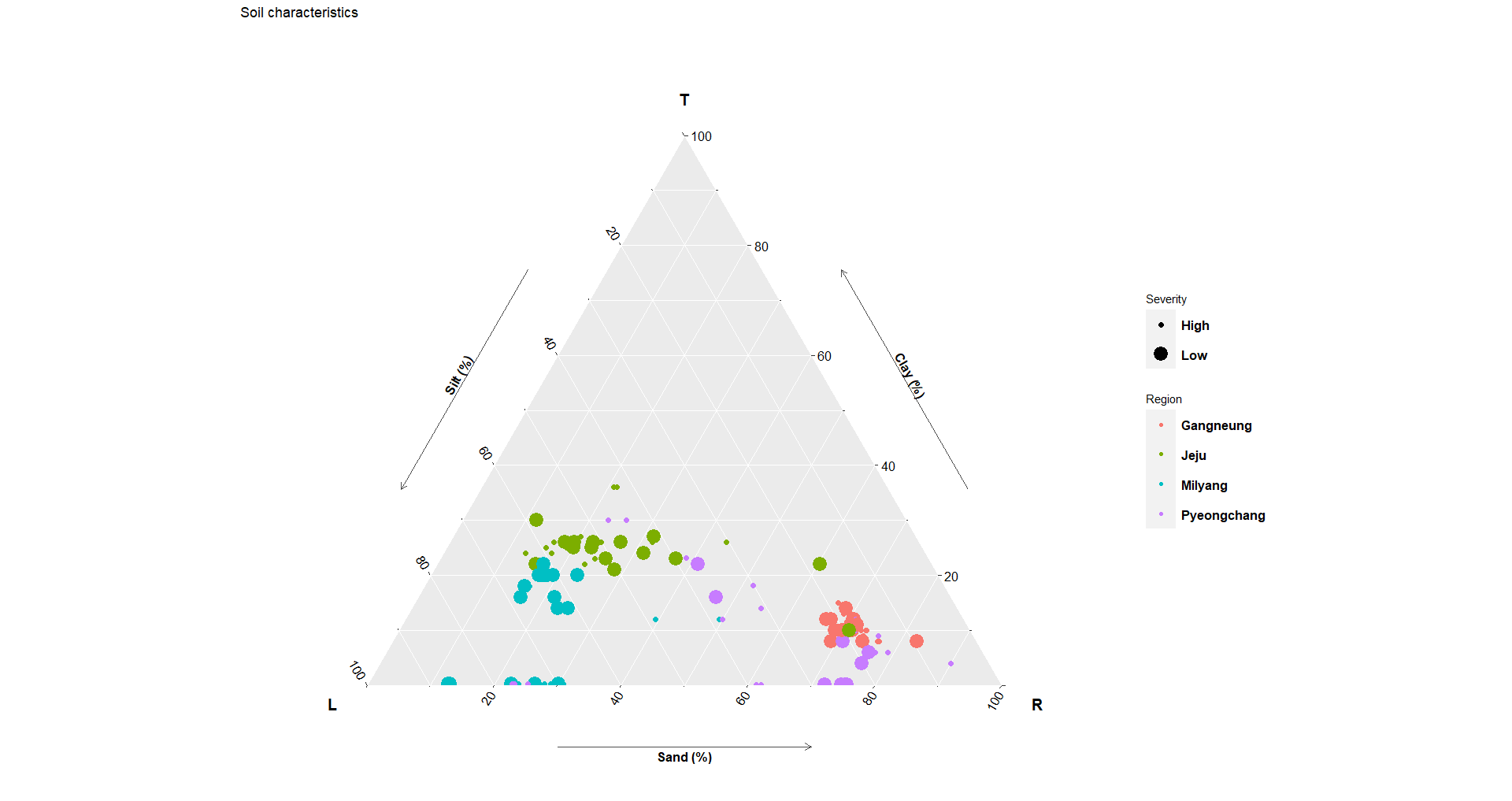


**sand**

**sandy loam**

**silt loam**

**sandy clay**

**loam**

**clay loam**

**silty clay loam**

**silty clay**

**sandy clay**

**clay**

**loamy sand**

**loam**


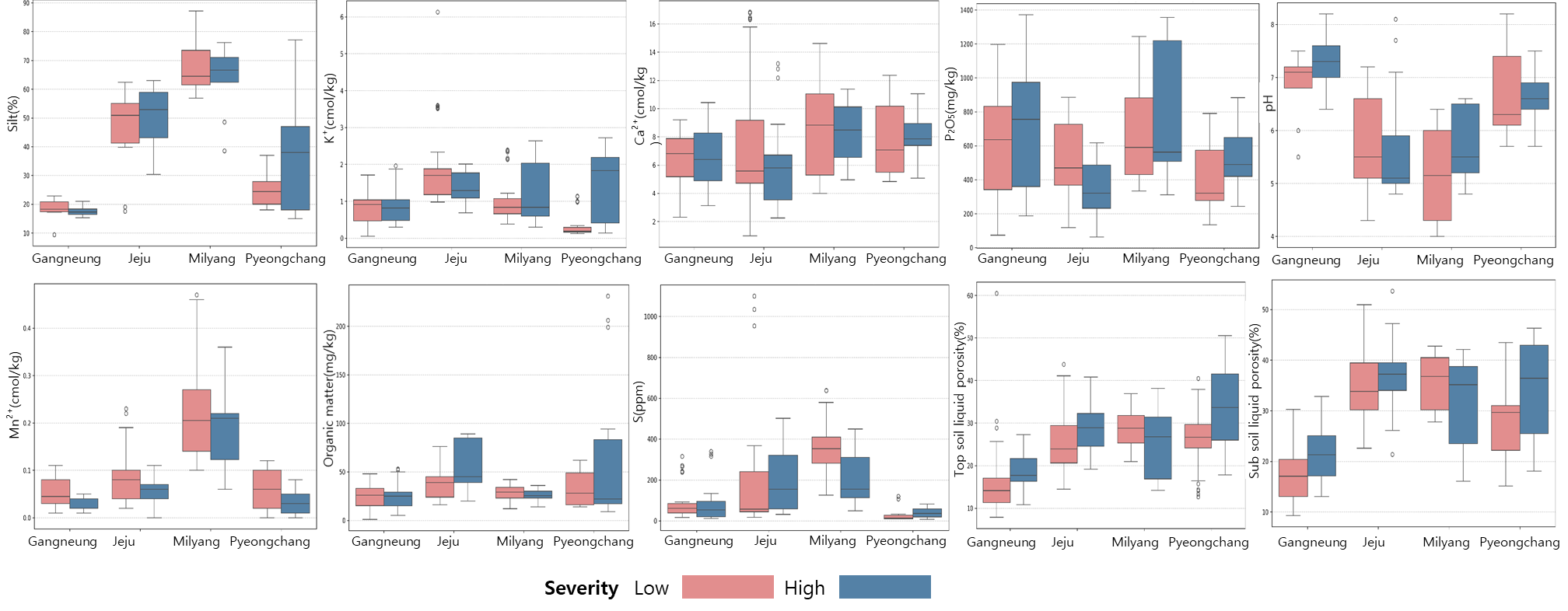


Supplementary Figure S3. Comparison of the top ten soil variables selected by the random forest model across different regions and potato common scab severity levels.


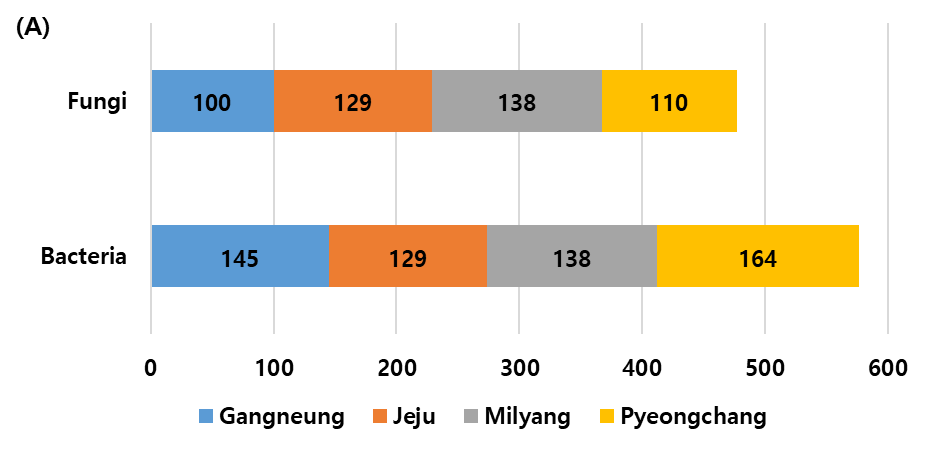

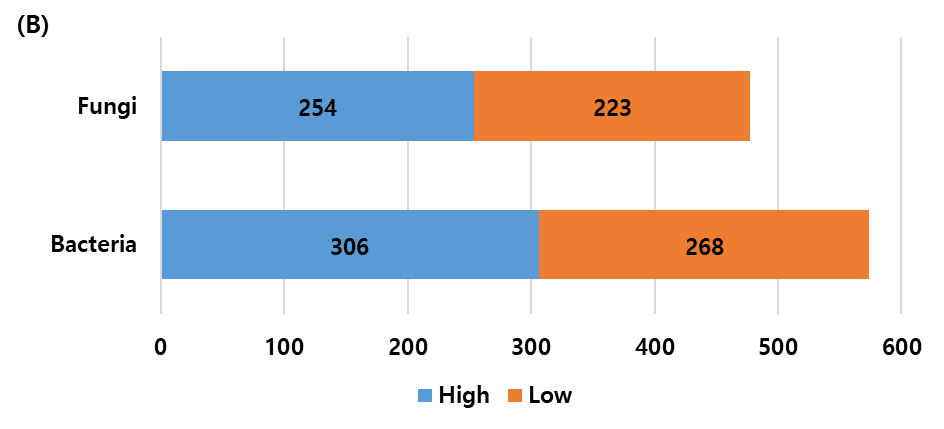


Supplementary Figure S4. Number of samples used for microbial community analyses classified by region (A) and potato common scab severity (B) for bacterial and fungal dataset.


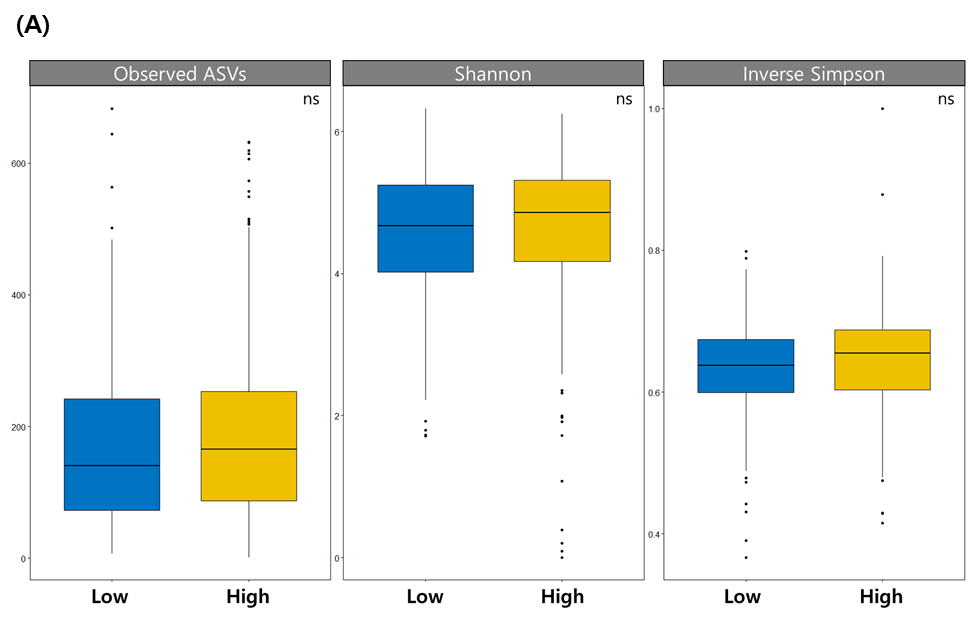

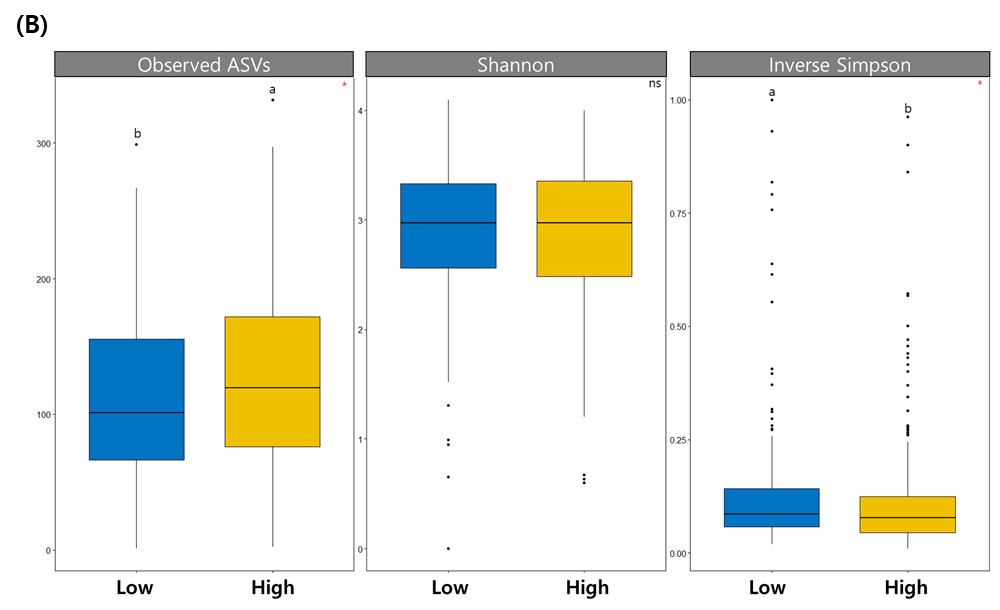


Supplementary Figure S5. Bacterial (A) and fungal (B) diversity according to potato common scab severity, Differences between two severity croups were evaluated using *t-test*, ns: no significant; * *p-value* < 0.05.


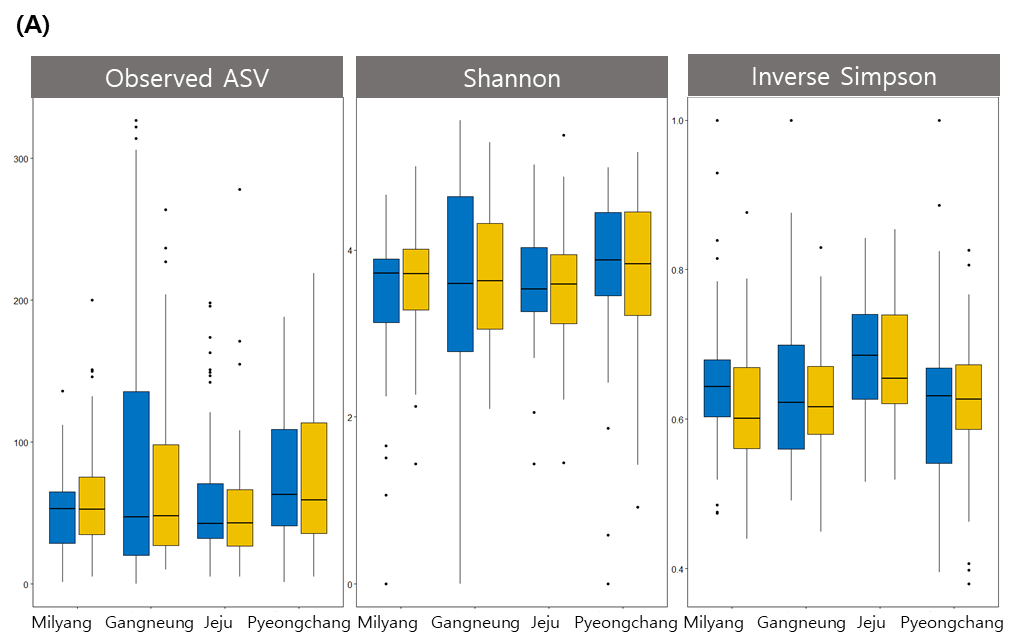

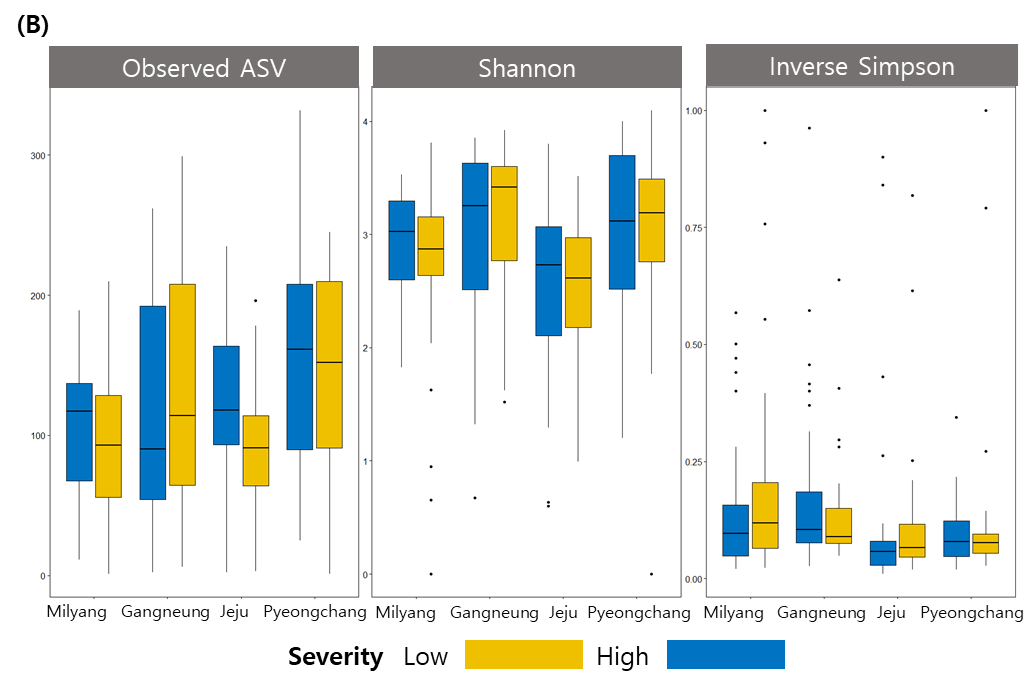
 Supplementary Figure S6. Bacterial (A) and fungal (B) diversity according to potato common scab severity in four different regions.


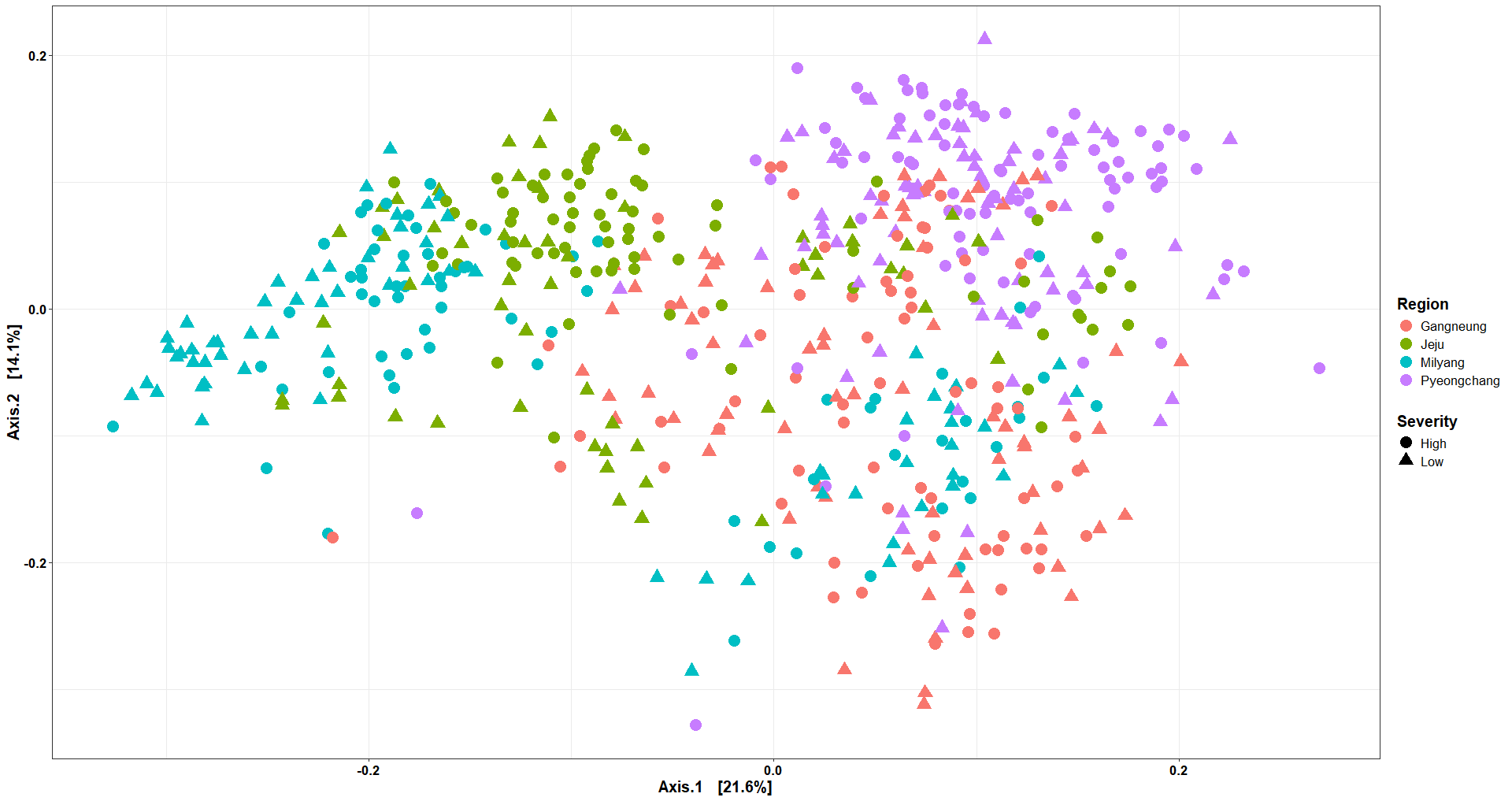


Supplementary Figure S7. Principal coordinates analysis (PCoA) of bacterial communities based on weighted UniFrac distance. Samples are colored by region and shaped by potato common scab severity.


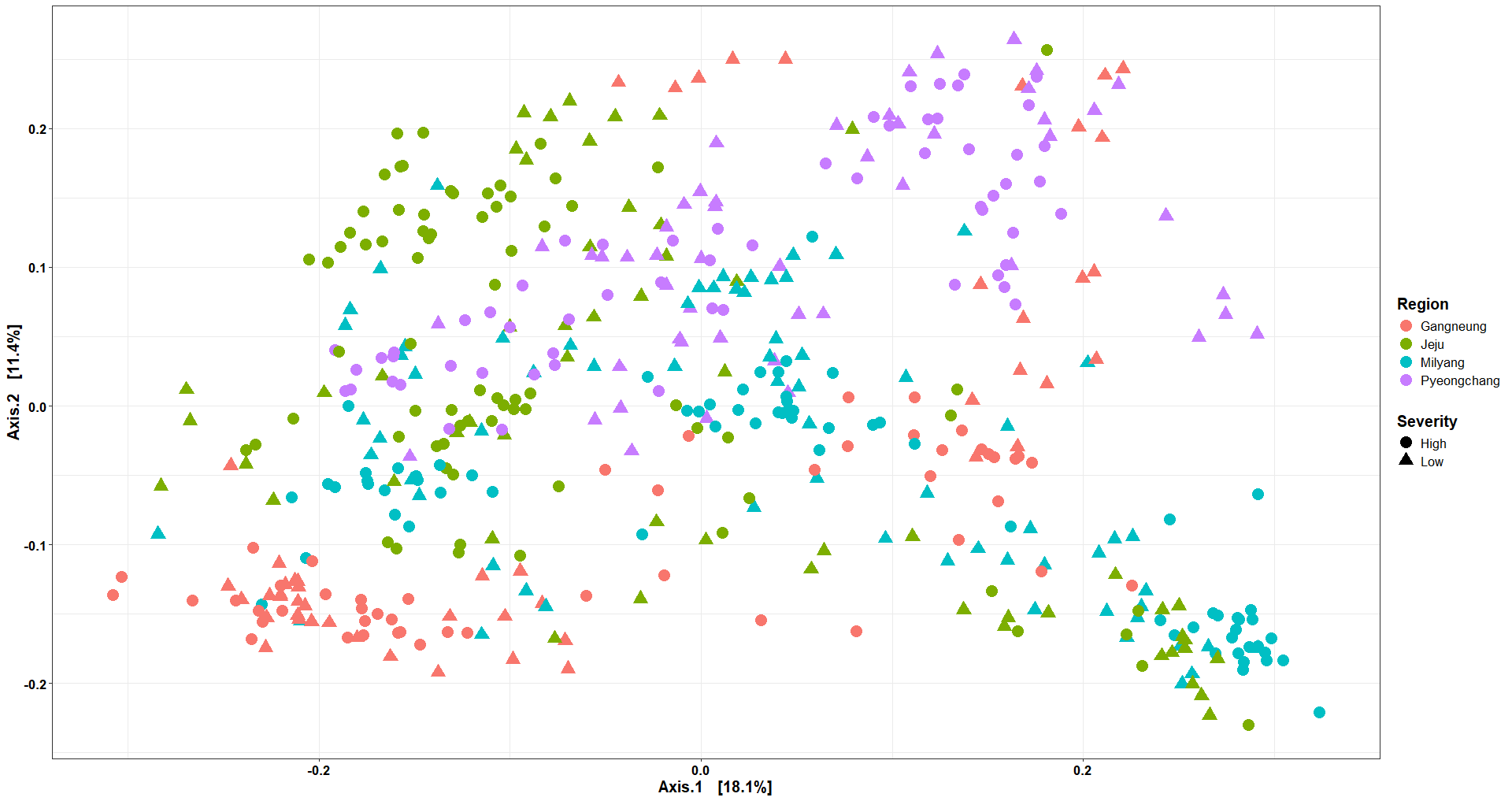


Supplementary Figure S8. Principal coordinates analysis (PCoA) of fungal communities based on weighted UniFrac distance. Samples are colored by region and shaped by potato common scab severity.


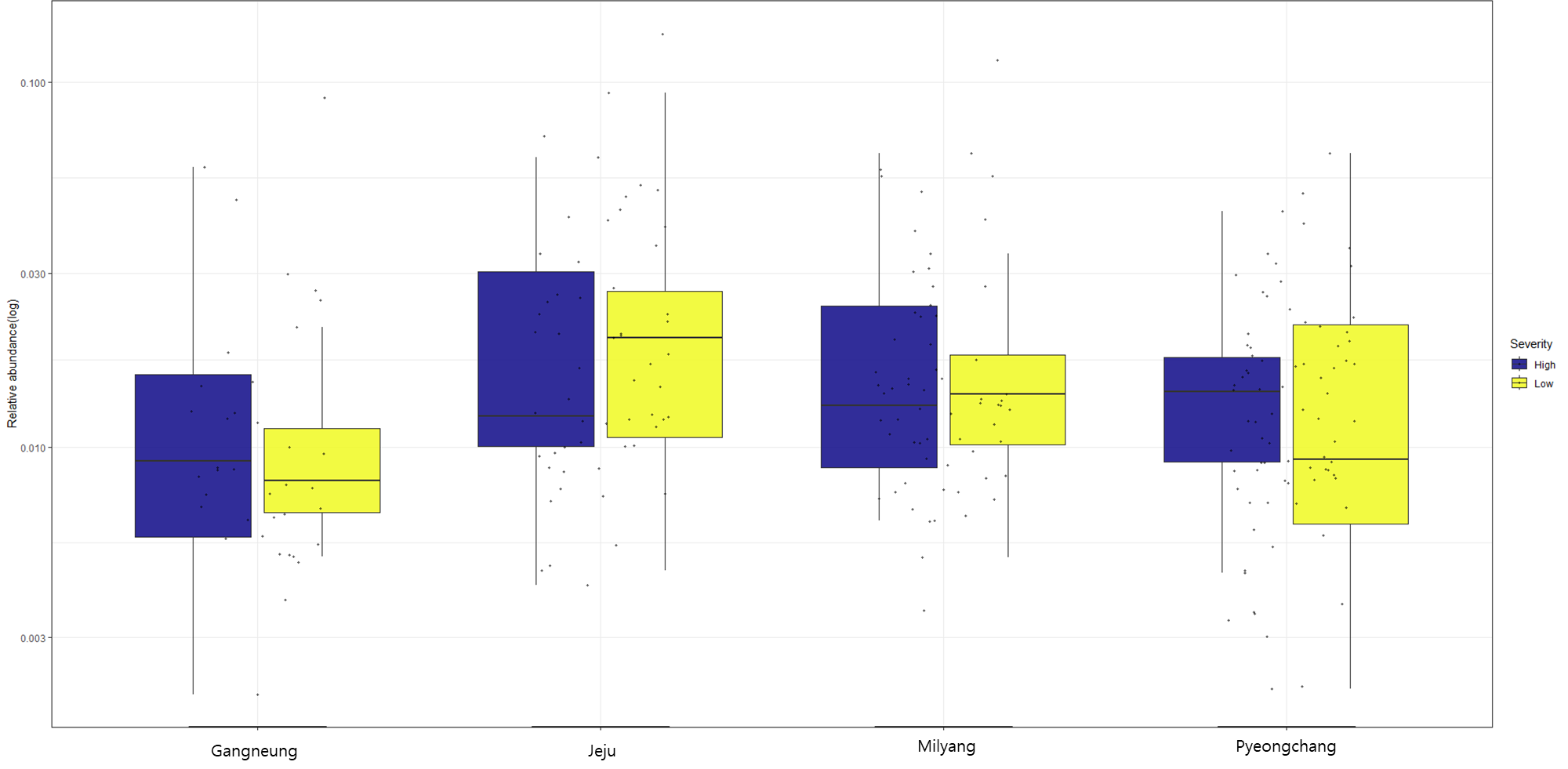


Supplementary Figure S9. Relative abundance of the Streptomyces genus across potato common scab severity groups. Boxplots represent the distribution of log-transformed relative abundance values from different regions.


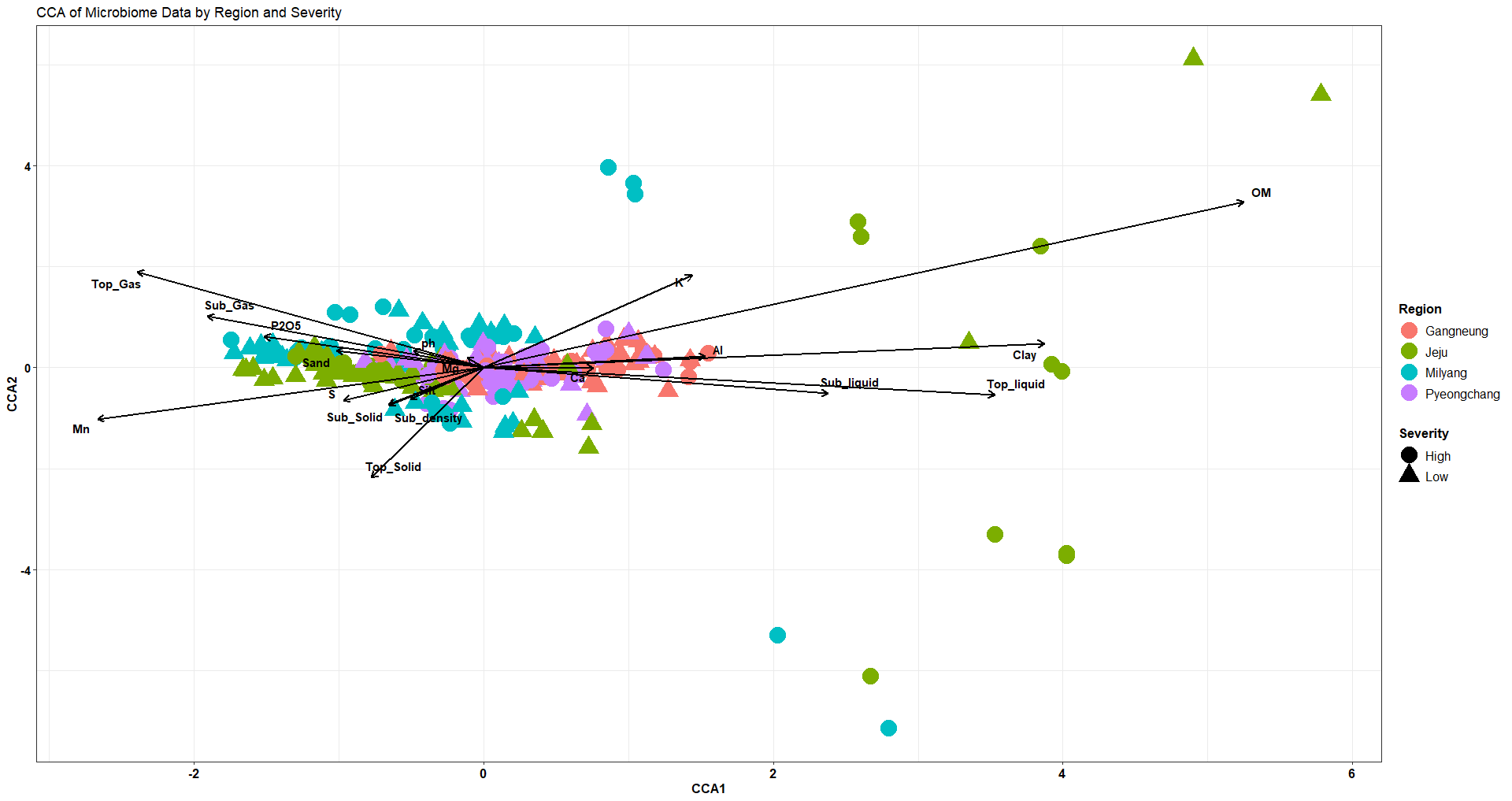


Supplementary Figure S10. Canonical correspondence analysis (CCA) of soil bacterial communities constrained by soil physiochemical properties. Arrows indicate soil physiochemical variables fitted to the ordination, with arrow length and direction reflecting the strength and direction of their relationships with bacterial community composition.


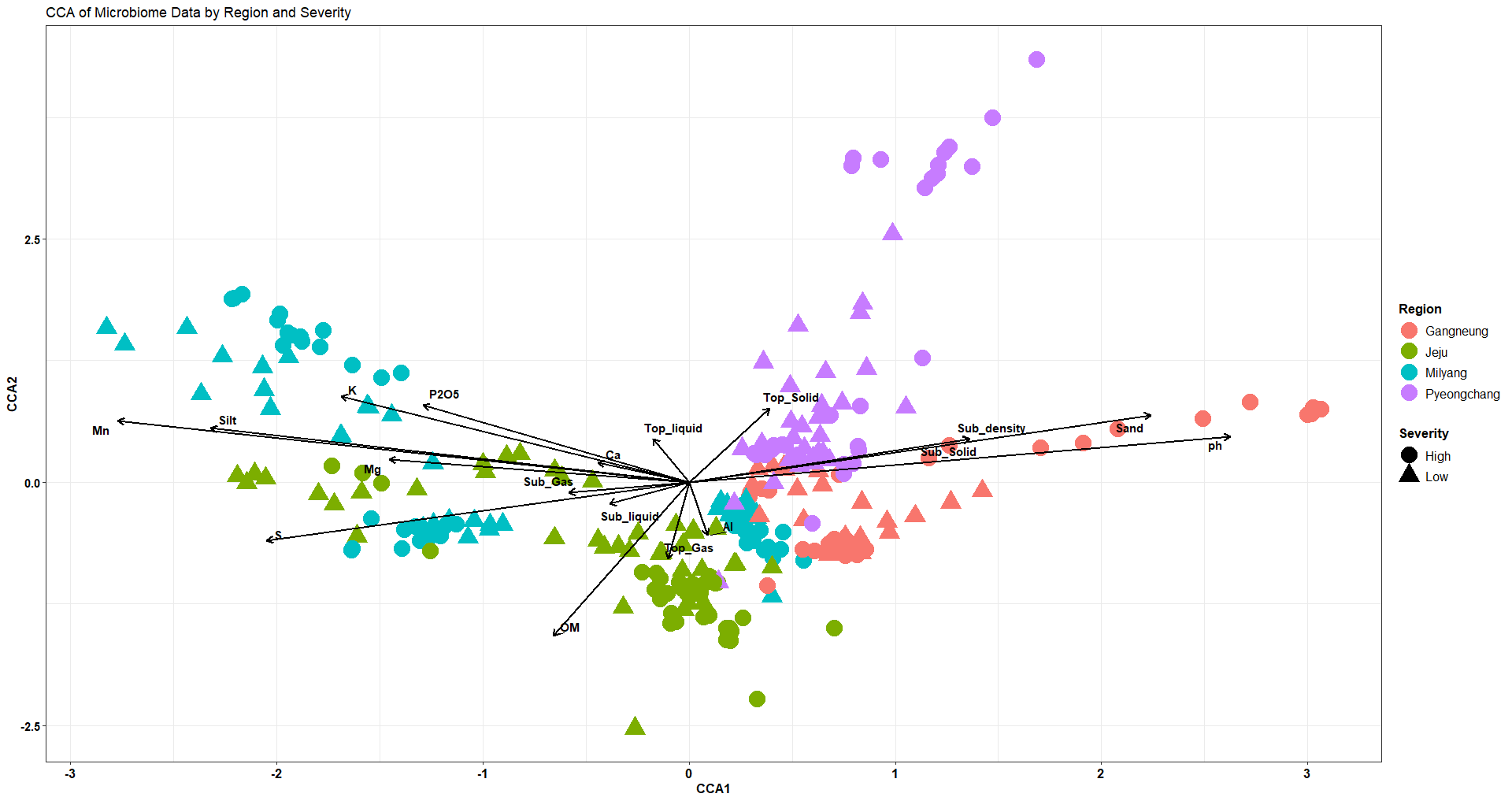


Supplementary Figure S11. Canonical correspondence analysis (CCA) of soil fungal communities constrained by soil physiochemical properties. Arrows indicate soil physiochemical variables fitted to the ordication, with arrow length and direction reflecting the strength and direction of their relationships with fungal community composition.

## Supplementary Table

Supplementary Table S1. Summary of ASV filtering steps and datasets used in downstream analyses.
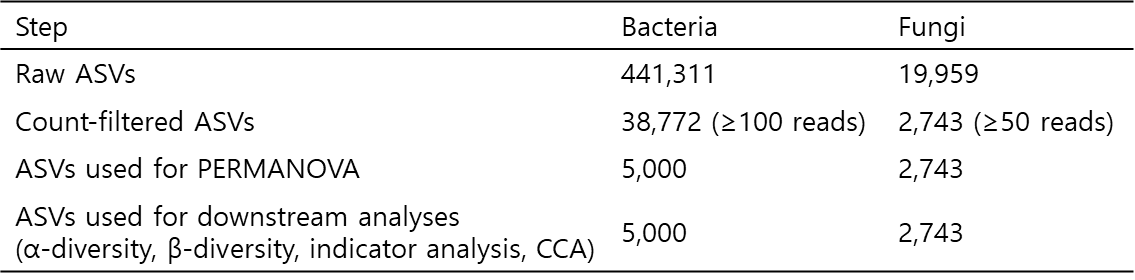

Supplement: Supplementary file 1 [file DataSheet1.docx]
